# IMatter: validation of the NHS Scotland Employee Engagement Index

**DOI:** 10.1186/s12913-014-0535-z

**Published:** 2014-11-08

**Authors:** Austyn Snowden, Ewan MacArthur

**Affiliations:** Institute of Mental Health, School of Health Nursing and Midwifery, University of the West of Scotland, Ayr, UK; School of Science, University of the West of Scotland, Paisley, UK

## Abstract

**Background:**

Employee engagement is a fundamental component of quality healthcare. In order to provide empirical data of engagement in NHS Scotland an Employee Engagement Index was co-constructed with staff. ‘iMatter’ consists of 25 Likert questions developed iteratively from the literature and a series of validation events with NHS Scotland staff. The aim of this study was to test the face, content and construct validity of iMatter.

**Methods:**

Cross sectional survey of NHS Scotland staff. In January 2013 iMatter was sent to 2300 staff across all disciplines in NHS Scotland. 1280 staff completed it. Demographic data were collected. Internal consistency of the scale was calculated. Construct validity consisted of concurrent application of factor analysis and Rasch analysis. Face and content validity were checked using 3 focus groups.

**Results:**

The sample was representative of the NHSScotland population. iMatter showed very strong reliability (α **=** 0.958). Factor analysis revealed a four-factor structure consistent with the following interpretation:

1. My experience as an individual
2. My experience with my direct line manager
3. My experience with my team
4. My experience with my organisation

Each subscale also showed high level of internal consistency within all disciplines. Rasch analysis confirmed the majority of items fit with the latent trait of staff engagement with infit statistics between 0.7 and 1.3; and showed a good spread of item difficulty covering person ability. Focus groups found the questionnaire valid it terms of brevity, relevance and clarity.

**Conclusions:**

iMatter showed evidence of high reliability and validity. It is a popular measure of staff engagement in NHS Scotland. Implications for practice focus on the importance of coproduction in psychometric development.

## Background

Employee engagement refers to the ‘*individual’s involvement and satisfaction with and enthusiasm for work*’ [1]. Employee engagement is associated with strong leadership, improved outcomes and in relation to healthcare has been defined as a positive parallel to burnout [2]. Where staff are engaged organisational performance is improved [3] and where staff are disengaged care fails [4].

In Scotland engagement currently sits at the centre of health strategy [5,6] and legislation [7]. However, despite a proliferation in tools designed to measure employee engagement fundamental problems persist with their validity and practical application. In NHS Scotland for example evidence of staff engagement has strongly relied on the ‘staff survey’ [8]. However, uptake is generally low, and staff attitudes to the survey are mixed. There is a sense of mistrust about the scope and purpose of the exercise. Therefore, in order to generate empirical data about staff engagement that would be trusted and valued by them, ‘iMatter’, an NHS Scotland Employee Engagement Index was co-produced with NHS Scotland staff between 2011 and 2013. This paper describes the validation process.

iMatter was initially developed from the policy and literature on staff engagement. The principles underpinning the individual items are embedded in The Quality Strategy [3], MacLeod Enablers [9], Knowledge and Skills Framework [10] and NHS Scotland staff governance standards [11]. The literature was utilised to ascertain existing measures of staff engagement and evaluate their strengths and weaknesses. Whilst some of these tools are clearly very well resourced and marketed [12,13], and others are already well validated [14,15] a fundamental weakness for the purpose of the current project was that all these ‘off the peg’ tools perpetuated a disconnect between the measurement process and the employees they were meant to represent. For example whilst many of the tools highlighted the importance of dialogue to engagement it was not evident that this had occurred during the *construction* of the tool itself. For example ‘communicating and involving your staff’ does not appear until page 72 of 83 in the NHS Employer’s (2011) staff engagement toolkit. The key element for the creation of any new tool designed to measure engagament was therefore the continuous engagement of the staff the tool was designed to measure.

### iMatter co-construction

As a starting point for developing iMatter an initial set of questionnaire items was drafted from a thematic analysis of the policy and theoretical literature in tandem with focus groups with NHS Scotland staff. The theory of staff engagement upon which the questionnaire was built therefore encompassed policy ideals, theoretical components of engagement [15] and most importantly the ‘real world’ operationalisation of these ideals and concepts as articulated by staff. The questionnaire was developed over a series of focus groups, pilot and validation events with NHS Scotland staff through 2011–2013. A sample of 160 NHS Scotland employees completed the first iteration of the questionnaire in pilot 1. A sample of 247 completed pilot 2 and a further 107 completed a third pilot. At each stage a series of focus groups were run with participating staff who made further recommendations to enhance face and content validity [16,17].

**Figure 1:**
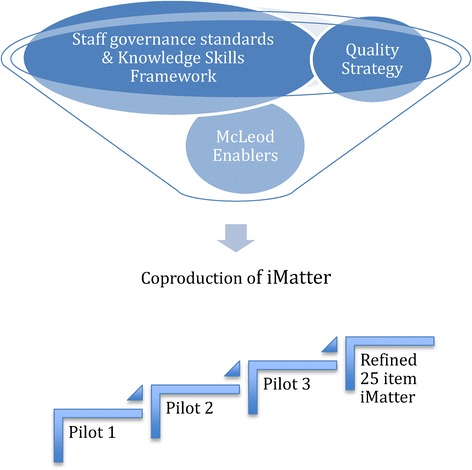
**Co-construction process of iMatter.**

Each iMatter item is therefore grounded in the policy, literature and experience of staff engagement and was co-constructed throughout with NHS Scotland staff (Figure 1). Scotland is the only country in the world to have developed such a systematic measure in this inclusive manner. This paper describes the psychometric validation of the final iteration of this questionnaire.

### Aim

The aim of this study was to validate iMatter in a nationally representative sample of NHS Scotland staff.

The objectives were:

1. To establish reliability of the scale,
2. To establish construct validity and factor structure,
3. To establish item difficulty and item fit,
4. To establish face and content validity.

## Methods

Construct validity was established with concurrent factor and Rasch analysis using a representative cross section of NHS Scotland staff. Face and content validity were checked using 3 focus groups with convenience sample of participants.

### Instrument

The version of iMatter tested here entailed the 25 Likert questions^a^ illustrated in Table 1. These questions were each followed by six Likert responses [*Strongly agree, Agree, Slightly Agree, Slightly Disagree, Disagree, Strongly Disagree*].

**Table 1:** **iMatter 25 items**

1	I am clear what my duties and responsibilities are
2	I get the information I need to do my job well
3	I am given the time and resources to support my learning and growth
4	I am confident my ideas and suggestions are listened to
5	I am confident my ideas and suggestions are acted upon
6	I feel involved in decisions relating to my job
7	I am treated with dignity and respect as an individual
8	I am treated fairly and consistently
9	I get enough helpful feedback on how well I do my work
10	I feel appreciated for the work I do
11	My work gives me a sense of achievement
12	I feel my direct line manager cares about my health and wellbeing
13	My direct line manager is sufficiently approachable
14	I have confidence and trust in my direct line manager
15	I am confident performance is managed well within my team
16	My team works well together
17	I would recommend my team as a good one to be part of
18	I understand how my role contributes to the goals of my organisation
19	I feel my organisation cares about my health and wellbeing
20	I have confidence and trust in senior managers responsible for the wider organisation
21	I feel involved in decisions relating to my organisation
22	I am confident performance is managed well within my organisation
23	I get the help and support I need from other teams and services within the organisation to do my job
24	I would recommend my organisation as a good place to work
25	I would be happy for a friend or relative to access services within my organisation

### Sample

In Jan and Feb 2013 iMatter was distributed both electronically and on paper to the total population of 2300 staff from three NHSScotland boards. All NHS staff from these boards was included and no staff was excluded. Demographic data entailed profession, team, hospital and organisation for sample descriptive and comparative purposes. The three focus groups post survey consisted of a convenience sample of 60 NHS staff from all participating disciplines and boards.

### Ethics

Because this was a research project involving NHS staff no NHS ethics approval was required. Permission to conduct the analysis was granted by the University of the West of Scotland ethics committee. The background information on the participant information sheet made it clear to staff that they were free to choose whether to participate or not, and that no recriminations would occur should they choose not to. No individual identifiable data was collected or requested.

## Results

### Sample descriptives

**Figure 2:**
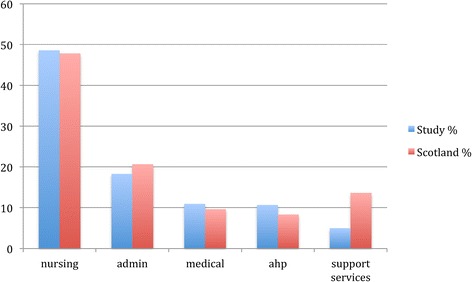
**Sample profession by % compared with national NHS Scotland workforce (admin = administration staff, ahp = allied health professionals).**

1280 people completed the questionnaire in total, a return of over 56%. For data cleaning purposes responses were removed where the questionnaire had only been partially completed. This left a sample of 1193. Full breakdown of the sample by profession is in Table 2. Although profession categories were measured slightly different from national statistics the full sample breakdown was comparable with the NHSScottish national workforce figures [18], suggestive of a nationally representative sample (Figure 2).

**Table 2:** **Respondents by profession**

**Response**					
		**Frequency**	**Percent**	**Valid percent**	**Cumulative percent**
Valid	medical	128	10.7	10.9	10.9
	nursing	569	47.6	48.6	59.5
	allied hp	125	10.5	10.7	70.2
	admin	182	15.2	15.5	85.7
	managerial	20	1.7	1.7	87.4
	support services	58	4.9	5.0	92.4
	corporate	12	1.0	1.0	93.4
	other	77	6.4	6.6	100.0
	Total	1171	98.0	100.0	
Missing	System	24	2.0		
Total		1195	100.0

### Responses

**Figure 3:**
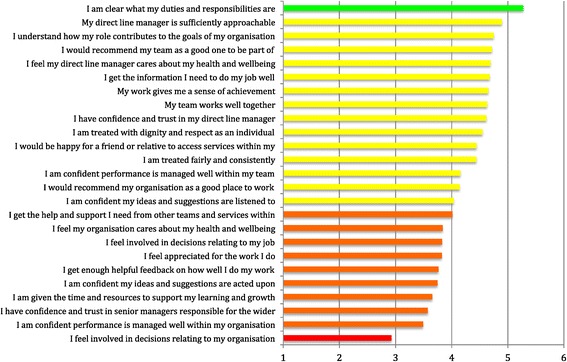
**Mean response to each iMatter item by rank (N=1193).**

Summary of mean responses to each question is in Figure 3. This shows that the most positive item by this ranking measure is ‘*I am clear what my duties and responsibilities are*’. The least positive is ‘*I feel involved in decisions relating to my organisation*’. In order to construct a summary measure individual scores for each item were added using the coding in Table 3. The total was then divided by the number of items answered. iMatter thus consists of an aggregate representation of all 25 individual item scores.

**Table 3:** **iMatter scoring**

**Score**	**Likert response**
1	Strongly disagree
2	Disagree
3	Slightly disagree
4	Slightly agree
5	Agree
6	Strongly agree

### Objective 1. Reliability

The 25 items had a very high level of internal consistency with Cronbach’s alpha of 0.958 utilising data from the full sample (N = 1193). This consistency was maintained regardless of profession (Table 4).

**Table 4:** **Internal consistency of iMatter in each profession**

**Profession**	**Number in sample**	**Cronbach’s alpha**	**Number of cases**
Medical	128	0.945	25
Nursing	569	0.957	25
Allied health professionals	125	0.96	25
Admin	214	0.955	25
Support services	58	0.957	25

### Objective 2. Factor analysis

A principal components analysis (PCA) was run on the full sample (N = 1193). The suitability of PCA was assessed prior to analysis. Inspection of the correlation matrix showed that all variables had at least one correlation coefficient greater than 0.4. The overall Kaiser-Meyer-Olkin (KMO) Measure of Sampling Adequacy was 0.946 with individual KMO measures all greater than 0.852, classifications of ‘meritorious’ to ‘marvelous’ according to Kaiser [19]. Bartlett’s test of sphericity was statistically significant (*p* < .000), indicating that the data was likely factorisable.

A principal component extraction of factors was conducted with a varimax rotation based on eigenvalues greater than 1. Coefficients below .4 were suppressed for ease of interpretation. This solution generated four factors although there was crossloading on 6 items (Table 5).

**Table 5:** **Factor structure of iMatter with loadings > .4**

**Rotated component matrix** ^**a**^				
	**Component**			
	1	2	3	4
I am clear what my duties and responsibilities are	.429			.595
I get the information I need to do my job well	.588			.473
I am given the time and resources to support my learning and growth	.651			
I am confident my ideas and suggestions are listened to	693			
I am confident my ideas and suggestions are acted upon	.720			
I feel involved in decisions relating to my job	.713			
I am treated with dignity and respect as an individual	.601		.462	
I am treated fairly and consistently	.612		.463	
I get enough helpful feedback on how well I do my work	.652			
I feel appreciated for the work I do	.643			
My work gives me a sense of achievement	.432			.403
I feel my direct line manager cares about my health and wellbeing			.820	
My direct line manager is sufficiently approachable			.853	
I have confidence and trust in my direct line manager			.828	
I am confident performance is managed well within my team			.528	.437
My team work well together				.803
I would recommend my team as a good one to be part of				.769
I understand how my role contributes to the goals of my organisation		.434		
I feel my organisation cares about my haealth and wellbeing		.670		
I have confidence and trust in senior managers responsible for the wider organisation		.784		
I feel involved in decisions relating to my organisation		.737		
I am confident performance is managed well within my organisation		.786		
I get the help and support from other teams… within the organisation to do my job		.647		
I would recommend my organisation as a good place to work		.707		
I would be happy for a friend or relative to access services within my organisation		.626		

Extraction Method: Principal Component Analysis.

Rotation Method: Varimax with Kaiser Normalization.

^a^Rotation converged in 12 iterations.

### Objective 3. Rasch analysis

Table 6 shows the item difficulty in the ‘measures’ column and the item ‘fit’ with the overall model as a function of infit mean square. Item 1 is therefore the easiest item by this measure (log odds −1.82) and item 21 the hardest (log odds 1.5). In relation to fit, items with an infit mean square close to 1 are optimal, although some variation is necessary [20]. Italicised items do not fit the model as well as expected.

**Table 6:** **Item difficulty and item fit for all 25 items**

**Entry**	**Measures**	**Infit Mean-square**	**S.E.**	**Labels**
1	−1.82	1.25	0.05	I am clear what my duties and responsibilities are
2	−0.59	0.92	0.04	I get the information I need to do my job well
3	0.73	1.22	0.03	I am given the time and resources to support my learning and growth
4	0.3	0.76	0.03	I am confident my ideas and suggestions are listened to
5	0.63	0.71	0.03	I am confident my ideas and suggestions are acted upon
6	0.54	0.87	0.03	I feel involved in decisions relating to my job
7	−0.39	0.91	0.04	I am treated with dignity and respect as an individual
8	−0.23	0.87	0.03	I am treated fairly and consistently
9	0.61	0.95	0.03	I get enough helpful feedback on how well I do my work
10	0.55	0.75	0.03	I feel appreciated for the work I do
*11*	*−0.55*	*1.48*	*0.04*	*My work gives me a sense of achievement*
*12*	*−0.61*	*1.37*	*0.04*	*I feel my direct line manager cares about my health and wellbeing*
*13*	*−0.97*	*1.68*	*0.04*	*My direct line manager is sufficiently approachable*
*14*	*−0.49*	*1.38*	*0.04*	*I have confidence and trust in my direct line manager*
15	0.15	0.93	0.03	I am confident performance is managed well within my team
*16*	*−0.51*	*1.42*	*0.04*	*My team works well together*
*17*	*−0.65*	*1.36*	*0.04*	*I would recommend my team as a good one to be part of*
18	−0.71	1.28	0.04	I understand how my role contributes to the goals of my organisation
19	0.53	0.82	0.03	I feel my organisation cares about my health and wellbeing
20	0.82	0.97	0.03	I have confidence and trust in senior managers responsible for the wider organisation
21	1.5	1.05	0.03	I feel involved in decisions relating to my organisation
22	0.91	0.81	0.03	I am confident performance is managed well within my organisation
23	0.32	1	0.03	I get the help and support I need from other teams and services within organization…
24	0.17	0.71	0.03	I would recommend my organisation as a good place to work
25	−0.23	1.03	0.03	I would be happy for a friend or relative to access services within my organisation

INPUT: 1192 Persons 25 Items MEASURED: 1192 Persons 25 Items 6 CATS.

#### Focus groups: face and content validity

Following completion of iMatter a sample of 60 multidisciplinary participants gave structured feedback on their thoughts about the tool in three focus groups. Main anxiety entailed issues of anonymity. Most other comments were very positive about clarity and brevity by comparison to other measures.

For example anxieties regarding anonymity persisted for participants from small teams. If people do not feel they can be genuinely anonymous then there remains a potential for disengagement with process. However, it was interesting to note that even these cautious individuals recognized and supported the necessity of the exercise. They suggested there may be a benefit to keeping paper copies in future iterations in order to mitigate any anxieties that may be related to perceived traceability when completing electronic versions.

Focus group participants were all very positive about the style and layout of questionnaire. They were particularly impressed with the short time it took, mainly in relation to comparable time-consuming questionnaires they had previously completed. Despite one or two comments about reducing the scale the six-point scale was broadly popular.

In summary, the focus groups provided positive feedback to support claims to face and content validity. They also provided further evidence of staff ‘buy in’ to the process. Even where specific criticisms were raised they were countered in every case by a stronger desire to complete the questionnaire. iMatter is a valid and *popular* measure of staff engagement in this sample.

## Discussion

**Figure 4:**
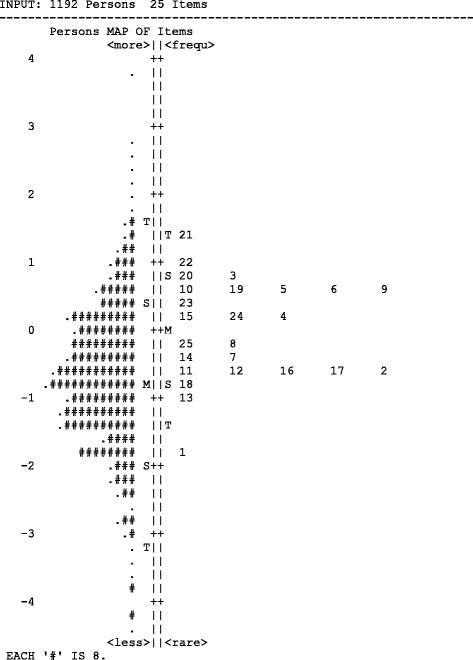
**Person ability and item difficulty.**

The principal component analysis generated four factors although there was crossloading on 6 items (Figure 4). Using the larger loading coefficient to generate an initial solution it can be seen that factor 1 entailed all the items except item 1 (2 to 11) from the section about ‘I/me’. Factor 2 entailed all the items about ‘my organisation’ (items 18 to 25). Factors 3 (items 12 to 14) and factor 4 (Items 1, 16 & 17) entailed the remaining questions. Four factors therefore best explained the principal component structure:

1. My experience as an individual
2. My experience with my organisation
3. My experience with my direct line manager
4. My experience with my team

These factors are therefore consistent with the purpose of the iMatter to measure these attributes. They also mirror the construction of the questionnaire in that these sections are essentially ‘subcategories’ of iMatter. The limitations of this interpretation will be discussed in more below. First however, in order to examine whether these factors were reliable in each profession Cronbach’s alpha was constructed for each subsample. The results are in Table 7 and demonstrate the reliability of this interpretation regardless of profession.

**Table 7:** **Chronbach’s alpha for each factor in each profession**

**Factor**	**Individual**	**Organisation**	**Manager**	**Team**
Medic (n = 128)	.918	.896	.895	.835
Nursing (n = 569)	.935	.898	.894	.803
AHP (n = 125)	.946	.898	.906	.728
Managers (n = 20)	.921	.867	.932	.699
Admin (n = 182)	.925	.905	.919	.778
Support (n = 58)	.928	.914	.927	.728

The questionnaire therefore represents measurement of a four factor structure. The credibility of this conclusion rests on the credibility of the theory underpinning the questionnaire. This may seem an almost trivial point, but it is important to stress that validation is a unitary construct that tests the theory and the measure at the same time [17]. Any conclusions that can be drawn from validation are a function of the conceptual strength of the underpinning theory [21]. Because iMatter was theoretically grounded, situated within policy, developed by staff and modified through a process of consultation over a series of robust cycles [Snowden and Macarthur, validation of the NHS Scotland Employee Engagement Index: Interim Report, Unpublished] the results of this study allowed trustworthy interpretations to be made.

The goal of factor analysis is to reduce the number of dimensions in the data (here 25 questions). Principal component analysis seeks linear combinations of variables that represent underlying fundamental quantities of which the observed variables are expressions. This results in a number of original factors that identify different aspects of the multidimensional data. These resulting constructs originate in the data and are not imposed at the beginning.

However, because the questionnaire was built from theoretical principles it would be hoped that factor analysis would show that the groups of items do in fact map on to these principles. If they do then the factor analysis has shown that the underpinning structure is consistent with the original theoretical understanding of staff engagement. The questionnaire measures what it is supposed to be measuring.

This is circular logic. The factors are almost bound to be interpreted as expressions of the principles because those principles drove the development of the questionnaire in the first place. This shows a limitation of using factor analysis in isolation and is why alternative interpretations are valuable. In this case Rasch analysis was utilised to deepen understanding of the performance of the items within questionnaire.

### Rasch analysis

In the Rasch model item difficulty is an expression of probability of getting a certain item ‘correct’. In this case it calculates the likelihood of a particular item within iMatter being positively endorsed. Item fit is a mathematical expression of how closely a particular item represents the underlying trait being measured. Rasch analysis tests the assumption that the items within a scale measure something of the same underlying trait, in this case staff engagement. It does this through an iterative process that generates an estimate of person ability, item difficulty and item fit [16]. This is done to simultaneously estimate:

- The likely response to a particular item according to the ability of the responding person and simultaneously: the likely ability of a person according to their item responses;
- The likelihood of a particular item fitting with the putative underlying trait.

These will be discussed in turn.

- *The likely response to a particular item according to the ability of the responding person and conversely the likely ability of a person according to their item responses*

The Rasch model tests all the items and all the people taking the test against each other at the same time. The starting point is to calculate the proportion of items answered correctly by each person, and the proportion of people successfully answering a particular item. These raw score totals allow an estimate of person ability and item difficulty. Person ability and item difficulty are sufficient to allow a calculation of the odds of success for a particular person on a particular item. These odds can be converted into a log scale, and converting odds into a log scale allows for standardisation of expression for item difficulty and person ability. This means they can be represented on the same scale. Figure 4 illustrates the sample according to person ability on the left and the difficulty of the individual items on the right. It shows that these items are a good test of ability for this sample given that the item difficulty spread is distributed within the sample ability.

There are few people unlikely to endorse the easiest question (item 1) and likewise there are very few people likely to need a more challenging measure of engagement than item 21. Because the item spread covers the person spread this means this questionnaire is a good measure of the entire range of engagement in this sample. The hardest question (item 24) is likely to be too hard to strongly agree with even by the most engaged people. Likewise the easiest question is still not easy for everybody. The Rasch analysis therefore showed that the questions that make up iMatter tap into a meaningful and diverse range of staff engagement. It is a useful measure across a range of abilities and thus a useful measure of change.

b) *The likelihood of a particular item fitting with the putative underlying trait*

Rasch analysis also assesses the degree to which all the items measure the same underpinning trait. This is called the latent trait in the Rasch literature [16] and the latent trait measured here is staff engagement. Infit mean square of one indicates the ideal model value of ‘staff engagement’. More or less than 1 indicates more variation than would be expected by the Rasch model. For example 1.3 is 30% more variation. 0.79 is 21% less variation. Response strings nearly always show some variation and this is no problem. In fact some variance is desirable in a multi item test. In the case of Rasch analysis variance of up to 30% is usually considered acceptable, although this reduces in very large samples [16]. Items 11, 12, 13, 14, 16 & 17 underfit the model according to this benchmark (italicised in Table 6). However removing these misfitting items would remove an entire section of the questionnaire. It would mean that items about ‘my direct line manager/my team’ are irrelevant to staff engagement, which is clearly not the case. Rather, this shows why benchmark values always need to be interpreted with caution and that multiple views should be considered in making decisions regarding item fit. These items need to be retained.

Item 11 is the most problematic item in terms of clear fit with either analysis. It is only moderately associated (.432) with factor 1 and therefore doesn’t add a great deal of extra information to ‘*my experience as an individual’*. It is also a poor fit with the Rasch model with an infit mean square of 1.48. However, it is a very interesting item in itself. As well as being mainly associated with factor 1 it is also moderately associated with factor 2 (.341) and factor 4 (.403) suggesting it contributes widely but not selectively to most factors. It is not easily pigeonholed, but ‘*my work gives me a sense of achievement*’ is important information to understand and should probably be retained.

### Synthesis

The rationale for using different statistical methods on the same dataset is that they facilitate different views. This is fundamentally useful as it gives a fuller picture than relying on one technique [22]. However, from a statistical perspective caution must be maintained when generating assumptions grounded in different measurements. On the positive side there is increasing credibility in combining Rasch analysis with factor analysis to study dimensionality, as done here [23]. In essence no single model can detect all possible sources of misfit and so it makes sense to view any dataset from multiple angles [24]. Factor analysis and Rasch analysis both provide evidence to support inferences regarding invariance within a particular context, and in this study they offered complementary explanations for the findings.

On the negative side these inferences are logically discrete. Rasch and factor analysis are embedded in different philosophical deconstructions of what constitutes measurement. Because of these different assumptions they are strictly speaking incompatible, mutually exclusive models [25]. However, Saltzberger goes on to state that

‘*proper scale development and analysis should never be confined to a statistical procedure (even if that procedure utilizes the Rasch Measurement), but should be guided by a theory of the construct to be measured’* ([25] p1376).

Rasch analysis and factor analysis help understand the data by viewing it in different ways and both are useful. A judgement does not need to be made on logical compatibility. It is not the techniques of data analysis that drive the credibility of the findings but the quality of the data and its underpinning theory. Where the raw data is of high quality it is reasonable to conclude that where Rasch and factor analysis illuminate similar patterns then these patterns are generalisable. Because the questionnaire was co-constructed with staff and embedded in policy and literature on employee engagement it is further reasonable to claim a credible interpretation of the psychometric analysis. A related strength of the study was the large and generalisable NHS Scotland staff sample. This means that any commonalities found are likely to be a function of generalizable population responses rather than psychometric artefacts.

The co-construction of the questionnaire has engendered and maintained a real sense of ownership by the staff, hence the favourable response rates. However, this local engagement probably comes at a cost of generalizability to other countries, given that it is likely to be the local ownership that has driven this engagement. In other words it is not the tool itself that is generalizable but the process of development. Other countries may not have the resources to construct their own measures.

## Conclusions

iMatter is a robust measure of staff engagement, meaningful and important to staff. The unique strength of this measure lay in its coproduction. The high response rate suggested that there was a genuine desire for staff voices to be heard and that they have endorsed this measure as a means to that end. For comparison the 56% response rate was more than double the 27% return of the 2010 NHSScotland staff survey, [18]. Participants must therefore remain involved in the feedback and evolution of iMatter in order to maintain this credibility.

In psychometric terms the Rasch analysis showed that most of the items fit with the latent trait of staff engagement. The factor analysis accounted for those items that did not appear to fit so well. Internal consistency was very high for the full scale and all the subscales in the whole sample and also in each subsample of professions. In this dataset this means that iMatter showed evidence of acceptable validity and reliability.

It remains to be seen if the high level of participation will continue as the project is further rolled out across Scotland. However, if it proves to be so then this paper has described a validation process that may help other countries follow suit in constructing a measure of engagement meaningful to their employees.

### Endnote

^a^The final version of iMatter entails *28* positively worded items. In the version tested here 3 of the 28 items were negatively worded. The purpose was to try to prevent people from providing homogenous responses. However this did not occur and further, the negatively worded items did not fit with the rest of the analysis. That is, not not feeling something is different from feeling it. The negative items were subsequently rephrased positively, in line with the remaining 25 items. This paper presents analysis based on the 25 positively worded items only.
